# Downregulation of RelA (p65) by Rapamycin Inhibits Murine Adipocyte Differentiation and Reduces Fat Mass of C57BL/6J Mice despite High Fat Diet

**DOI:** 10.1155/2014/540582

**Published:** 2014-01-23

**Authors:** Peter D. Ray, Reid A. Maclellan, Jin He, Zhigang Liu, Jianguo Wu

**Affiliations:** ^1^Department of Surgery, Division of Plastic Surgery, University of Alabama at Birmingham, 1670 University Boulevard, VHG94, Birmingham, AL 35294, USA; ^2^Boston Children's Hospital, Department of Plastic and Oral Surgery, Boston, MA 02115, USA; ^3^Renmin Hospital of Wuhan University, Department of Anesthesiology, Wuhan, Hubei 430060, China

## Abstract

Rapamycin (RAPA) is a clinical immunosuppressive agent first reported in the literature in 1975 after its discovery in a soil sample from the island of Rapa Nui. Aside from the well-documented effects of RAPA on cell division and immunologic response, the literature reveals it to have negative effects on adipocyte and osteocyte differentiation as well. Understanding of the molecular effects of RAPA on cell differentiation is fragmentary in regard to these cell lineages. In this paper, we examined a potential mechanism for RAPA's effects on adipocyte differentiation *in vitro* and *in vivo*. The data point to a unique role of Rel A (p65)—a component of the NF-*κ*B system—in mediating this event. In murine adipose derived stem cell cultures (muADSCs) from C57BL/6J mice, RAPA was found to selectively downregulate RelA/p65, mammalian target of rapamycin (mTOR), and do so in a dose-dependent manner. This implies a novel role for RelA in adipocyte biology. Intracellular lipid accumulation—as subjectively observed—was also decreased in muADSCs treated with RAPA. Mice treated with RAPA had reduced overall body weight and reduced size of both intraabdominal and subcutaneous fat pads. When treated with RAPA, mice fed a high fat diet did not develop obesity and were not different from their regular diet controls in terms of body weight. These results suggested that RAPA inhibits adipogenesis and lipogenesis of muADSCs resulting in a prevention of obesity in C57BL/6J mice. This inhibition is strong enough to negate the effects of a high fat diet and seems to act by downregulating the RelA/p65 mTOR signaling pathway—a key component of the NF-*κ*B family.

## 1. Introduction

In the 1950s molecular geneticists were trying to determine if proteins built from amino acids were required for the synthesis of nucleic acid chains or vice versa [1]. As a result of this work, the Rel gene was described in *E. coli* in 1969 [2] and so named because when the gene was present it rendered “relaxed” mutants of *E. coli* more stringently dependent on the presence of specific amino acids for successful RNA synthesis. “Rel proteins” share genetic homology and yet initiate different proliferative and/or transformative effects on different cells [3]. The first described “Rel protein” (REV-T) was most intriguing because of its ability to transform cells into malignant populations [4]. A viral form (v-Rel) was then also identified in birds, implying that there existed a related homology across species of these regulator proteins [5]. Due to their ability to drive replication and transformation from bacteria to mammals, the Rel genes were studied extensively in an attempt to understand their relation to malignancy [6, 7].

Further investigation to identify proteins related to c-Rel identified a number of subunits which were found to exist bound to each other as dimers or heterodimers. This led to the concept of a “Rel family of proteins,” meaning a set of proteins that regulate transcription and possess the structural ability to form complexes required for DNA binding. Rel family proteins are often described as part of a broader Rel/NF-*κ*B transcription system [8].

Much research has been done on the NF-*κ*B system since its discovery by Sen more than 25 years ago [9]. The NF-*κ*B family members form complexes, combining as dimers from five possible subunits: p50, p52, c-Rel, RelB, and/or RelA (p65). Of these, the first two (p50 and p52) have motifs that contain nuclear localization sequences. The other Rel family members are the transcriptionally active components that exist in the cytoplasm of a cell bound to an inhibitor of Kappa B (I*κ*B) [10].

NF-*κ*B/Rel protein translocation is generally understood to occur by two distinct pathways: canonical (conforming to well-established patterns) and noncanonical (unique pattern). The canonical pathway, which is triggered by inflammatory stimuli, controls innate immunity and protects hepatocytes, lymphocytes, and macrophages from programmed cell death. It involves NF-*κ*B1 (p50). As mentioned previously, an inhibitor, I*κ*B, masks the nuclear localization sequence as a baseline. This masking retains inactive NF-*κ*B dimers, usually p50/p62, in the cytoplasm. Engagement of receptors on the cell surface activates phosphorylation of I*κ*B by its kinases (inhibitor of kappa B kinases or IKKs). I*κ*B degrades as a result and releases the dimer for nuclear translocation.

The alternate (or noncanonical) NF-*κ*B pathway, driven by NF-*κ*B2, operates at the interface of innate and adaptive immunity, meaning that it is intimately involved when fighting off foreign cells [11]. The exclusive partner of NF-*κ*B2 p100 and p52 is RelB. NF-*κ*B2 activation is regulated by NF-*κ*B inducing kinase (NIK), which appears to have an obligatory function in immune cell differentiation based on studies from knockout models [12–14]. In the alternate noncanonical pathway, phosphorylation of NF-*κ*B2 (p100) is mediated by IKK*α* and NIK to trigger release of p52-RelB dimers for nuclear translocation [15].

What is unknown, however, is the importance of the Rel A component of the NF-*κ*B system as it has not been shown to either induce malignancy or contribute to immune response. Given the clinical finding of poor wound healing in human transplant patients taking the drug RAPA (personal observation), we hypothesized that there may be a mechanism at work on the nonimmunologic level (as a side effect) which impaired subcutaneous tissue regeneration. We hypothesized that an impairment of stem cell differentiation may play a role and found that in the mouse model the RAPA effect involved the Rel A segment of the NF-*κ*B system.

The mammalian target of rapamycin (mTOR) is at the crossroads of a nutrient-hormonal signaling network that is involved in specific pathological responses, including obesity [16]. mTOR forms two distinct signaling complexes known as mTOR complex 1 and mTOR complex 2 (mTORC1 and mTORC2). mTORC1 and mTORC2 contain shared and unique partners, distinct substrates and have differing sensitivities to rapamycin, the molecular functions of which remain poorly understood [17]. mTORC1 is sensitive to rapamycin and promotes anabolism while inhibiting catabolic cellular processes. Growth factors and nutrients promote mTORC1-dependent protein synthesis, cell growth, and metabolism [18]. Conversely, insufficient levels of these factors, or signals of cell stress, rapidly suppress mTORC1 and mTORC1—controlled anabolic processes [19, 20]. Reduced mTORC1 signaling also activates autophagy, a degradative process that may represent the last line of defense against starvation [19–21]. In contrast, mTORC2 contains exclusively rictor (rapamycin-insensitive companion of mTOR), mSin1 (mammalian stress-activated map kinase-interacting protein 1), and PRR5/protor (protein observed with rictor 1 and 2). Rictor and mSin1 promote mTORC2 assembly and signaling; the function of PRR5/protor remains obscure [22].

The clinical immunosuppressive agent RAPA is used extensively to prevent graft rejection in transplant patients [23]. Early studies show an inhibitory effect of RAPA on adipocyte glucose uptake [24] and differentiation associated with mTOR pathway [25]. In view of the remarkable effect of brief RAPA treatment in causing loss of both subcutaneous and intra-abdominal fat as well as protecting against high fat diet-induced obesity in C57BL/6J mice, we strove to identify the subunit responsible for its effects.

Here, we show that RAPA selectively downregulates RelA/p65 and inhibits adipogenic differentiation of murine mesenchymal stem cells derived from adipose tissue. The consequence of muADSC exposure to RAPA is inhibition of NF-*κ*B signaling pathway and downregulated expression of mTOR. In contrast, c-Rel and Rel-B were not changed. These findings suggest a practical avenue for antiobesity therapy and insight into mechanisms of wound healing or disrupted regeneration regulated by RelA/p65 expression in mammals. It may explain why transplant surgeons frequently complain about patients who are on RAPA having wound healing problems.

## 2. Materials and Methods

### 2.1. Animals

The normal mice (C57/BL6) used in this study were purchased from The Jackson Laboratory (Bar Harbor, ME). The Institutional Animal Care and Use Committee approved all animal protocols used for these experiments.

### 2.2. Cell Harvest and Culture

For isolation of murine adipose-derived stem cell cultures (muADSC) cells, omental (OM) and subcutaneous (SUB) adipose tissue was excised separately from ten C57/BL6 mice/group (6 months of age) with/without rapamycin and with/without high fat diet. The samples were finely minced and digested using 0.075% collagenase type XI (Sigma, St. Louis, MO) for 45 min at 37°C. Enzyme activity was neutralized by treatment with Dulbecco's modified Eagle's medium (DMEM) containing 10% fetal bovine serum (FBS). The cell suspension was centrifuged at 1200 g for 10 min to separate the floating adipocytes from the stromal vascular faction (SVF). The SVF was resuspended in ammonium chloride (160 mM), incubated at room temperature for 10 min to lyse contaminating red blood cells, and then centrifuged as described above. After filtration of the cell suspension through a 100 mm nylon strainer to remove cellular debris, cell number and viability were determined using trypan blue exclusion. The SVF cells were plated in T75 flasks overnight in control medium (DMEM, 10% FBS, and 1% penicillin/streptomycin) at 37°C in 5% carbon dioxide (CO_2_). After incubation for 24 h, the flasks were washed extensively using phosphate buffered solution (PBS) to remove residual nonadherent cells. The adherent muADSC cells were expanded using serial passaging.

### 2.3. *In Vitro* Adipogenic Differentiation Assay

Cell monolayer from basal cultures was expanded in standard growth medium (DMEM + 10% FBS) to ensure mid-log growth phase confluence (60 to 80%). Medium and floating cells from the culture flask were aspirated and discarded. DPBS was added (using 5 to 10 mL). The cell monolayer was then gently rinsed. After removal of the DPBS, 5 to 7 mL of prewarmed TrypLE Express was added to the flask and until the culture surface was completely coated. The cell monolayer was then incubated for 5 to 8 minutes at 37°C or until cells had fully detached. The detached cells were then pipetted into a single cell solution and verified on an inverted microscope. The cell suspension was removed from the flask, transferred into a centrifuge tube, and spun at 100 ×g for 5 to 10 minutes.

Cell viability and total cell density were confirmed using Trypan Blue Stain. The pellet was resuspended in an appropriate volume of culture medium. The muADSC cells were then replated in 6-well plates (100,000 cells per well) in control medium to allow attachment. Twenty-four hours later, the medium was replaced with new control medium or adipose medium (control medium plus insulin (10 mM), dexamethasone (1 mM), isobutyl-methylxanthine (0.5 mM), and indomethacin (200 mM) (all from Sigma-Aldrich)). Cultures were maintained for 14 days, and refed every 3 to 4 days. The cultures then were assessed using Oil Red O stain, which serves as an indicator of intracellular lipid accumulation. The cells were fixed for 10 min at room temperature in 10% neutral buffered formalin and were washed with distilled water. They then were incubated in Oil Red O (Millipore, Temecula, CA) reagent for 30 min and washed 3 times with distilled water. The cells were counterstained with hematoxylin for 1 min and examined under microscope.

### 2.4. Characterization of muADASC Cells

Flow cytometry was used to characterize muADSC cells obtained from C57/BL6 mice with/without HFD (high fat diet) or with/without rapamycin and grown for 2 passages and 4 passages under control conditions. Cultured cells were trypsinized, spun, and washed in cold PBS 1X (Mediatech, Herndon, VA) containing 2% FBS. The cells then were divided into aliquots and were spun to form a pellet. Cells were blocked using mouse serum (Sigma) diluted 1 : 10 in PBS and rat anti-mouse CD14/CD31 (BD PharMingen, San Diego, CA) for 10 min on ice. The primary antibodies (applied in optimal amounts) included a biotin-conjugated rat anti-mouse monoclonal antibody against CD34 and CD29 followed by streptavidin-APC, PE-conjugated mouse anti-Sca-1, and FITC-conjugated mouse anti-CD45 and anti-CD44 (all from BD PharMingen, San Diego, CA). Analysis of surface protein expression was performed using appropriate gating on viable CD45-negative cells to eliminate contaminating hematopoietic cells. The isotype antibody control samples obtained for each individual cell population were used to set the dot-plot intercepts used for the analysis.

### 2.5. Western Blotting

Cells were lysed in 1% NP-40 lysis buffer (1% NP-40, 150 mM NaCl, 50 mM Tris-HCl (pH 8.0), and 1 mM sodium orthovanadate) supplemented with protease inhibitors (0.1 mM PMSF, 2 *μ*g/mL each of leupeptin and aprotinin) for 15 min on ice. Total cell lysates were clarified by centrifuging at 10,000 rpm for 5 min at 4°C. The Pierce Protein Assay was used to estimate protein concentrations, with BSA as the standard. Equivalent amounts of cell lysates (20 *μ*g of total protein) were loaded onto 4 to 20% SDS-PAGE gradient gels, transferred onto nitrocellulose membranes, and probed with the appropriate antibodies. Western blot analysis was carried out according to standard procedures using ECL detection (Amersham, Piscataway, NJ), using antibodies reactive to NF-*κ*B family proteins, and DR4 (Santa Cruz Biotech, Santa Cruz, CA). Changes in protein level was determined through densitometry using Adobe Photoshop and normalized for loading against the actin as appropriate.

### 2.6. Data Analysis and Statistics

Statistical significance was determined using the InStat computer program (GraphPad Software Inc., San Diego, CA).

## 3. Results

### 3.1. Phenotypic Characterization of the muADSC Cells

The visceral adipose tissue from 1 mouse yielded approximately 7.0 × 10^6^ nucleated cells. Within 4 passages after the initial plating of the primary culture, muADSC cells appeared as a monolayer of large, flat cells. As the cells approached confluence, they acquired a spindle-shaped or fibroblast-like appearance. We used flow cytometry to evaluate the expression of CD45, Sca-1, CD34, CD44, and CD29 cell-surface antigens on the muADSC cells obtained from C57/BL6. The muADSC cells were negative for CD45, a hematopoietic cell surface marker. Approximately 50% and 10% of the muADSC cells were Sca-1 and CD34 positive, respectively, at the time of isolation (data not shown). As shown in Table 1, muADSC cells were positive for CD29, CD44, CD73, and Sca-1 and were negative for CD14, CD31, CD45, and CD34 at the time of flow cytometry analysis (after 4 passages).

### 3.2. muADSC Cells Undergo Adipogenic Differentiation *In Vitro*

**Figure 1:**
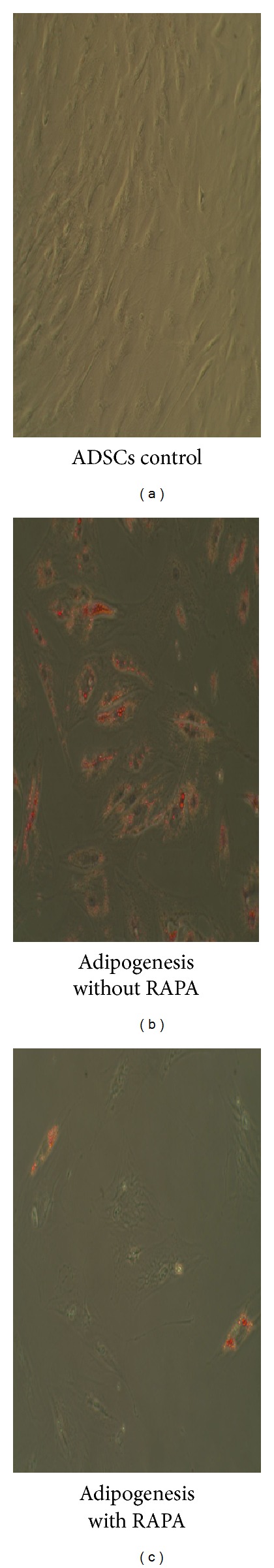
Rapamycin (RAPA) inhibited adipogenesis of ADSCs *in vitro*. (a) Resting cells in control media (CM) showed a differentiated contractile phenotype characterized by an irregular shape. (b) Incubation with adipogenic differentiation medium for two weeks induced adipocyte differentiation. Oil Red-O staining of differentiated muADSC without rapamycin treatment show significant numbers of cells and robust phenotype. (c) Differentiating muADSC cells treated with rapamycin and stained with Oil Red-O demonstrated visibly fewer cells and a smaller phenotype.

To determine whether muADSC cells undergo adipogenesis, we cultured muADSC cells in adipogenic medium. Oil Red O staining performed 2 weeks later revealed cytoplasmic lipid droplets, which are indicative of the adipogenic phenotype. These results indicate that the muADSC cells cultured in adipogenic medium underwent adipogenic differentiation (Figure 1(b)), whereas muADSC cells maintained in control medium did not (Figure 1(a)).

### 3.3. Effects of RAPA on Body Weight in LFD- and HFD-Induced Mice

**Figure 2:**
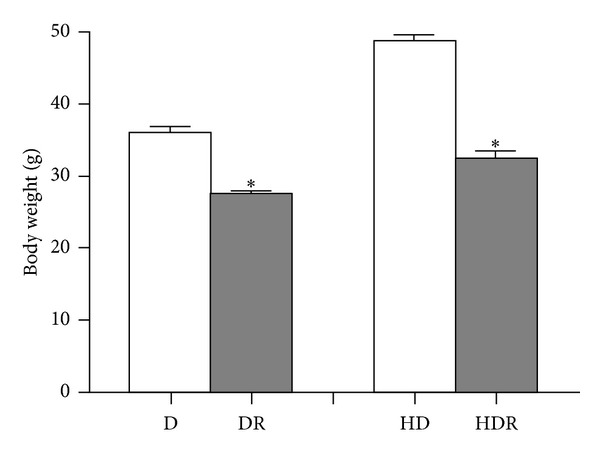
Rapamycin (RAPA) reduced body weight of C57BL/6J mice on both high fat and low fat diets. D = 10% fat diet; R = rapamycin; HD = 40% high fat diet; data are expressed as mean ± S.E.M. *n* = 10 mice for each group. *P* < 0.001 (*).

However, surprisingly, RAPA-treated mice had reduced body weight on either the low fat diet (LFD) or high fat diet (HFD) compared to the LFD or HFD control mice. The most marked difference was observed after 1 week of RAPA treatment at 6 months of age when a 8.5 g mean difference (24% weight lost) for LFD-fed mice with/without RAPA and 16 g of mean difference (33% weight lost) for HFD-fed mice with/without RAPA (*N* = 10 each group) in body weight was measured (35.95 ± 0.9293 versus 27.45 ± 0.5843 in LFD-fed mice and 48.73 ± 0.7200 versus 32.66 ± 0.5837 in HFD-fed mice, *P* < 0.001) (Figure 2).

### 3.4. Effects of RAPA on Fat Pads Mass on the LFD/HFD

The Sub and OM weights of RAPA-treated mice were significantly lower (*P* < 0.001) than those of control mice fed on the LFD or HFD (Table 2). When normalized for body weight, RAPA treatment significantly (*P* < 0.001) reduced Sub by 65% and OM by 72% in RAPA-treated mice with LFD and 47% and 56% in RAPA-treated mice with HFD, respectively. These findings indicated a reduction in fat pad weights and that RAPA prevented the effect of diet on the rate of accretion in fat pad weight.

### 3.5. Effect of RAPA on Adipocyte (ADSCs) Differentiation

When omental or subcutaneous preadipocytes in primary culture were induced to differentiate into adipocytes, the presence of RAPA severely impaired the adipogenesis. Very few cells show the characteristic morphology of rounded, lipid-filled adipocytesunder microscopic visualization as shown in Figure 1. The RAPA-mediated inhibition of adipocyte differentiation was observed to be almost identical in omental or subcutaneous tissue samples.

### 3.6. Effect of RAPA on the Expression of p65/RelA, mTOR, and the Adipocyte Differentiation Markers in ADSCs Differentiation

**Figure 3:**
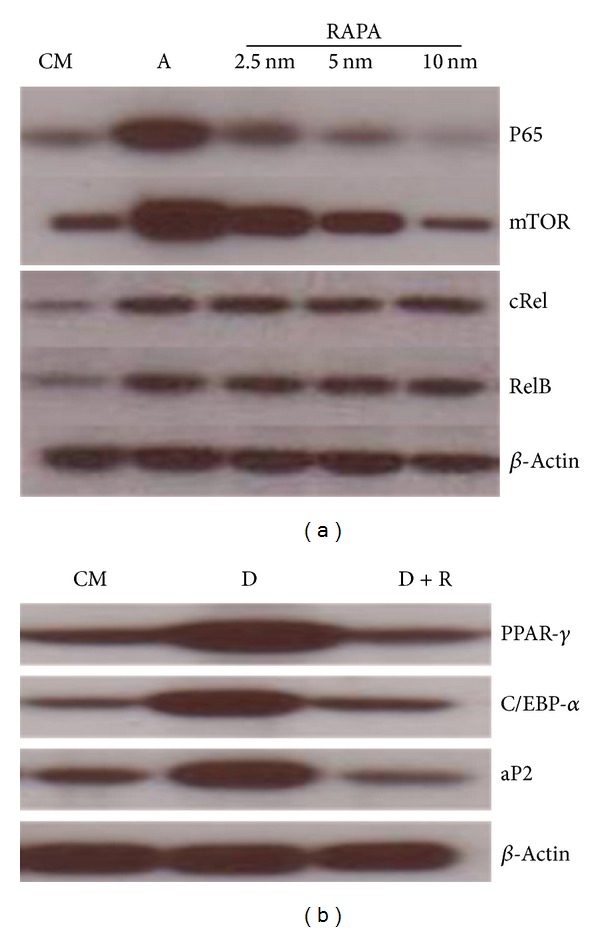
(a) RAPA inhibited the expression of p65/RelA and mTOR during adipocyte differentiation in a dose-dependent manner. Increasing amounts of RAPA were added to muADSC cell culture groups to determine dose dependency. After 6 days posttreatment, total proteins were extracted and Western blot analysis was performed with specific antibodies to RelA, RelB, c-Rel, and mTOR. The antibody to *β*-actin was used for loading control. (b) RAPA represses the expression of adipogenic marker genes such as PPAR-gamma, C/EBP-*α*, and aP2 during adipocyte differentiation. 10 nM RAPA was added into muADSC cell cultures. After 6 days posttreatment, total proteins were extracted and Western blot analysis was performed with specific antibodies to PPAR-gamma, C/EBP*α*, and aP2. Expression of *β*-actin was used for loading control. The results are representative of three independent experiments.

RAPA treatment led to a decrease of p65/RelA, mTOR, *α*P2, C/EPB*α*, and PPAR-gamma in differentiated ADSCs cells and is dose dependent (Figure 3(b)). ADSCs at baseline were assessed by Western blot analyses. p65/RelA, mTOR, *α*P2, C/EPB*α*, and PPAR-gamma were strongly induced in *day 6* differentiated ADSCs compared with undifferentiated ADSCs (Figure 3, lines 2 and 1). Our data suggest that this decrease may contribute to the inhibited adipogenesis in RAPA-treated adipocytes.

## 4. Discussion

In this study, we examined the effects of rapamycin (RAPA) on obesity of the C57BL/6J mouse strain fed with either low or high fat diets. Results support our prediction that a high fat diet significantly promotes the development of obesity [26] and the treatment of RAPA can reduce body weight gain of mice fed with HDF. The loss of body fat (both omental and subcutaneous) we observed in RAPA-treated mice may be due to a decrease in average adipocyte size or as a result of reduced formation of new adipocytes from precursor cells [27]. The activation of mTORC1 signaling in 3T3-L1 adipocytes by ectopic expression of Rheb inhibits expression of ATGL (adipose triglyceride lipase) and HSL (hormone-sensitive lipase), suppresses lipolysis, increases *de novo *lipogenesis, and promotes intracellular accumulation of triglycerides [28]. Inhibition of mTORC1 signaling by rapamycin or knockdown of raptor stimulates lipolysis primarily *via* activation of ATGL expression [29]. Those findings demonstrate that mTORC1 promotes fat storage in mammalian cells by suppression of lipolysis and stimulation of *de novo *lipogenesis. These result are consistent with RAPA-reduced expression of most adipogenic marker genes including PPAR-gamma, C/EBP*α*, FAS, *α*P2, adipsin, and ADD1/SREBP1c, all affecting lipogenesis and adipogenesis [30]. Thus, the ability of RAPA to prevent obesity suggests that RAPA or a molecular derivative without immunologic side effects might be a valuable tool to modulate key energy balance regulators.

Our data also indicate that rapamycin impairs omental and subcutaneous adipocyte differentiation in primary culture. Rapamycin inhibited the characteristic accumulation of intracellular lipid, as well as the expression of *α*P2, PPAR-gamma, and C/EBP*α*, the specific markers of adipogenesis. The molecular basis underlying this regulation of RAPA associated mTOR signaling pathway is not fully understood. Rapamycin, upon binding to FKBP12, interacts with mTOR, which then leads to the inhibition of p70 S6 kinase [31]. Recent findings reveal that mTOR itself is a serine kinase. It is capable of autophosphorylation, as well as the phosphorylation (and activation) of p70 S6 kinase. Rapamycin, then, may inhibit p70 S6 kinase as a consequence of blocking the mTOR serine kinase activity. Because insulin is an adipocytedifferentiating hormone and p70 S6 kinase is known to participate in insulin signaling, it was postulated that rapamycin blocked adipogenesis by attenuating insulin signal transduction and blocking critical postconfluent mitoses in the early phase of adipogenesis [32]. We have shown here that rapamycin is effective in attenuating mouse adipocyte differentiation. Both omental and subcutaneous preadipocytes responded similarly to rapamycin, suggesting that preadipocytes derived from either of these anatomic regions use similar insulin signal transduction pathways to trigger adipogenesis.

The precise rapamycin-sensitive pathway regulating adipocyte differentiation remains to be defined. It is possible that the loss of subcutaneous fat that results when humans are given subcutaneous fat injections of glucocorticoids is acting in a similar manner [33]. Insulin is believed to stimulate the mTOR kinase and its phosphorylation of p70 S6 kinase [34], but others have instead reported that p70 S6 kinase phosphorylation is increased indirectly through the inhibition of a mTOR-regulated phosphatase [35]. In addition, mTOR appears to be located upstream of several other signaling proteins in addition to p70 S6 kinase. These include 4EBP-1/PHAS1, a regulator of mRNA translation and cell proliferation, and the cyclin-dependent kinases [36]. Multiple signaling pathways regulated by mTOR may therefore be operative in the preadipocyte.

In summary, we have demonstrated that RAPA dramatically reduced the expression of p65/RelA and inhibited differentiation of ADSCs. At the same time, RAPA revealed no effects on cRel or RelB in our experiments. Our studies provide new insight into the current understanding of the biological function of NF-*κ*B, especially the p65/RelA, in adipocytes differentiation. Targets downstream of mTOR by modulating p65/RelA should be the focus of future studies on the regulation of adipogenesis.

## Figures and Tables

**Table 1:** Phenotypic characterization of muADSC cells. FACS analysis of muADSC demonstrating expression of typical surface proteins. Cultured cells used in the experiments were uniformly positive for CD29, CD44, CD73, and Scal-1 and negative for CD14, CD31, CD34, and CD45.

General surface markers for MuADSC at passage 4	
Positive (+)	Negative (−)
CD29	CD14
CD44	CD31
CD73	CD34
Sca-1	CD45

**Table 2:** Effects of rapamycin on fat weights in C57BL/6J mice fed with D (10% fat diet) or HFD (40% fat diet), with or without rapamycin (R). Data are expressed as mean ± SEM. *n* = 10 mice for each group. *P* < 0.001. Sub: subcutaneous; OM: omental.

	D	DR	HD	HDR
Absolute weight (g)				
Sub	1.18 ± 0.054	0.34 ± 0.022**	1.87 ± 0.068	0.66 ± 0.051**
OM	1.45 ± 0.089	0.24 ± 0.029**	1.88 ± 0.109	0.55 ± 0.043**
Percent of body weight (%)				
Sub	3.26 ± 0.102	1.25 ± 0.064**	3.83 ± 0.106	2.02 ± 0.146**
OM	3.69 ± 0.240	0.87 ± 0.091**	3.85 ± 0.177	1.68 ± 0.123**

Data are presented as means ± SEM. The two-tailed *P* value is <0.0001 (**), extremely significant.
